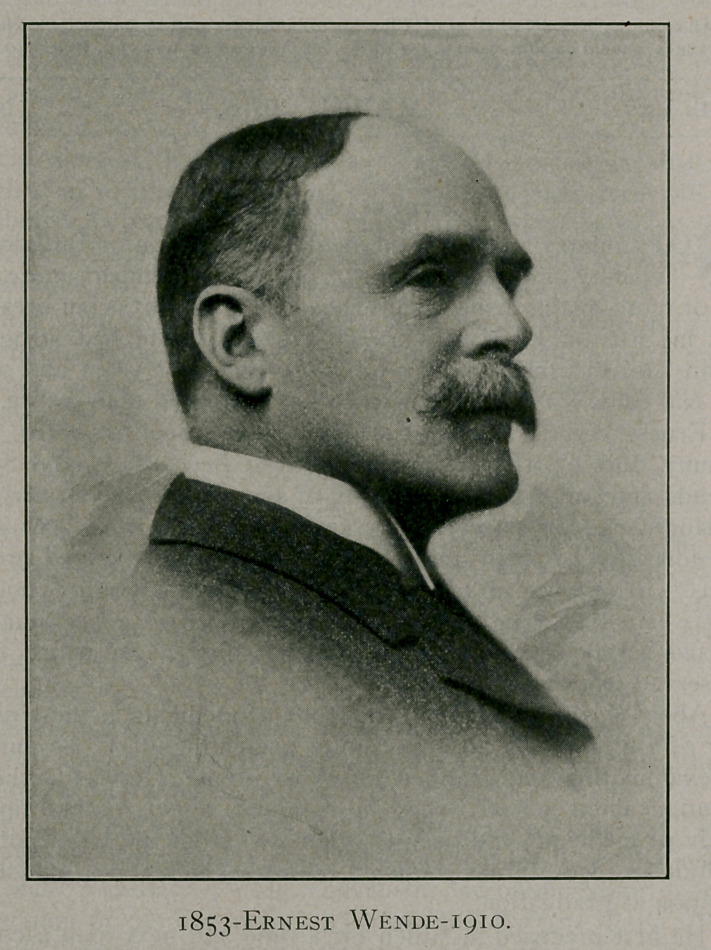# Ernest Wende

**Published:** 1910-03

**Authors:** 


### BUFFALO MEDICAL JOURNAL.

**A Monthly Review of Medicine and Surgery.**

*EDITOR*

WILLIAM WARREN POTTER, M. D.

All communications, whether of a literary or business nature, books for review and xchanges, should be addressed to the editor, 238 DELAWARE AVENUE, BUFFALO, N.Y.

VOL. LXV. MARCH, 1910. No. 8

### Ernest Wende

NOT in many years has the medical profession of Buffalo and vicinity suffered the loss of one of its members more useful or more distinguished than when this great physician breathed out his life on Friday, February 11, 1910. He had struggled against an incurable malady for more than two years and when the end came it must have been to him a blessed deliverance.

Ernest Wende was born at Mill Grove, town of Alden, Erie County, July 23, 1853. He was a son of Bernard A., and Susan Wende and the eldest of a family of ten children, nine sons and a daughter. His parents were farmers. They were bent upon the education of their children and most of them entered professions. Ernest was graduated from the Buffalo High School in 1874. He then taught school at Alden for two years and was elected a school commissioner for that district, in which office he served two terms.

About this time he began the study of medicine at the University of Buffalo, but, an opportunity presenting itself, he entered an examination and qualified for West Point. He was at the institution about a year, when he resigned, again to take up the study of medicine, and he was graduated from the University of Buffalo in 1878. For a thesis on influenza, he received honorable mention at graduation.

He first practised at Alden for a time and next took a course in the College of Physicians and Surgeons, now the medical department of Columbia University. In 1884 he began a course at the University of Pennsylvania, and the following year received the degree B. Sc. He then went to Europe, where he took special instruction in skin diseases and microscopy at Berlin and Vienna. He returned to Buffalo in 1886 to take up the practice of medicine as a life work.

He was almost immediately appointed clinical professor of diseases of the skin at the University of Buffalo, to succeed Professor Kellicott. He was also appointed professor of botany and microscopy of the Buffalo College of Pharmacy.

From the very beginning Dr. Wende took a high place among the teachers at the University of Buffalo devoting himself solely to teaching and to the practice of dermatology, his chosen specialty, until January 1, 1892, when he was appointed health

1853-Ernest Wende-1910.

commissioner by Charles F. Bishop, then mayor, under the new charter of Buffalo just going into effect. Five years later he was reappointed by General Edgar B. Jewett, then mayor. After another five years Dr. Walter D. Greene, who had been deputy commissioner during Dr. Wende's second term, was appointed to the chief place, whereupon Dr. Wende retired to private life. When, however, five years more had passed and Hon. J. N. Adam became mayor, Dr. Wende was recalled to the office which he held until his death.

During the thirteen years that Dr. Wende held the office of health commissioner of Buffalo, he instituted many reforms and improvements some of which have been adopted by other cities. To mention a few we may call attention to the thorough inspection of the source of the city's milk supply, strict methods in regard to the quarantine of contagious diseases, bacteriological and chemical examination of the water supply, inspection of the markets, hotels, restaurants and like places, where careless or purposely shiftless preparation of food has often been the cause of disease and death. While these would be included in a partial list of improvements effected during Dr. Wende's regime as health commissioner, yet we think his greatest achievement was in the abolishment of the long-tube nursing bottle. Soon after taking office the first time, Dr. Wende found the infantile death rate unconscionably high and began an investigation as to its cause. He discovered at once depressions on the inner walls of the tubes which afforded lodgement to bacteria that infected the milk. After a long struggle, during which the druggists vigorously opposed him, he succeeded in obtaining the passage of an ordinance abolishing the long-tube bottle, and immediately the high death-rate among infants sank to a very low figure. He demonstrated these imperfect and dangerous tubes at meetings of the Medical Society of the State of New York, of the American Public Health Association, and before other bodies. Many other cities followed in Dr. Wende's lead and soon the long-tube nursing bottle became almost unknown. Mothers learning its deadly effect established a boycott against it and the problem was solved.

Dr. Wende was a member of the Medical Society of the County of Erie, of the Medical Society of the State of New York, of the American Dermatological Association, of the American Microscopical Society, and of the Pan-American Medical Congress. He was a Fellow of the Electro-Therapeutic Association, and of the Royal Microscopic Society of England. He was at one time vice-president of the American Public Health Association. He also was prominent among local social and fraternal associations. He had been for long years supreme president of the Order of Iroquois in this city. He took an active part in the formation of the Municipal League and was its active head for several years. He was associate editor of the BUFFALO MEDICAL JOURNAL from 1895 until his end.

From this brief but imperfect sketch it will be seen that Ernest Wende was a strong character, a man of forceful impulses, and an original thinker. He wielded a vigorous pen and has contributed many articles of value and importance to medical literature. He was a man of many accomplishments, as instanced by his studies in ethnology and archeology; by his knowledge of botany, which he taught with rare acumen, and, as might be inferred by his fondness of rod and gun. Again, he was a sanitarian of rarely gifted skill and knowledge, as was demonstrated by his service as health commissioner, which we have enlarged upon already.

But, first and foremost, Ernest Wende was a physician, a specialist, a dermatologist. He was gifted as a diagnostician in an especial degree and often was called upon for an expert opinion in obscure or doubtful cases. His success in treatment, too, must not be forgotten, for here indeed, he possessed great skill.

He is survived by his mother, who still lives at the old family home in Mill Grove; his wife, who was Miss Frances H. Cutler of Omaha, Neb.; three children, Flavilla Frances, Margaret Winifred and Hamilton Heath Wende; by his brothers, Dr. Horatio S., of Tonawanda; Drs. Grover W. and John Wende, Bernhard P., Frederick of New York, and Albert Wende, now in the Cobalt region, and one sister, Miss Minnie Louise Wende.

The funeral was held at the home, 471 Delaware Avenue, on Sunday, February 14, 1910, and burial was at Forest Lawn. Seldom have such distinguished honors been paid the memory of one of our citizens as on this occasion. More than 500 of Buffalo's leading men and women visited the Wende home in the afternoon and about 300 remained for the funeral service. They included city officials, lawyers, doctors, prominent citizens and business men who knew and admired Dr. Wende.

Reverend George B. Richards, rector of the Church of the Ascension, read the short and simple Episcopal service. There was no singing, and after the prayers those who were still waiting outside were admitted.

The bearers were: Dr. H. R. Hopkins, former Mayor Charles F. Bishop, who appointed Dr. Wende in 1892; Police Commissioner W. D. Doherty, Judge Louis P. Hart, former Mayor J. N. Adam, former Mayor Dr. Conrad Diehl, Dr. P. W. Van Peyma, Dr. C. G. Stockton, Dr. Charles Cary and Dr. W. G. Gregory.

The active bearers were all officials of the health department: August Schneider, Secretary, Drs. T. B. Carpenter, W. G. Bissell, F. C. Gram, W. H. Heath, F. B. Willard, F. E. Fronczak, Deputy Commissioner of Health, John Rast and Stephen W. Bateson.

At a special meeting of the Medical Society of the County of Erie, held at the University Club, Saturday, February 12, 1910, a select committee was appointed to represent the society at the service, composed of the following named members:

Conrad Diehl, Henry R. Hopkins, Charles G. Stockton, A. H. Briggs, Charles Wall, Edward Clark, Francis E. Fronczak, John Petit, Walter D. Greene, F. Parke Lewis, Elmer Starr, Roswell Park, Matthew D. Mann, R. R. Ross, Franklin C. Gram, DeLancey Rochester, Harry Mulford, James S. Smith, Arthur W. Hurd, Thomas B. Carpenter, Charles Cary, Carlton R. Jewett, Herman E. Hayd, Earl P. Lothrop, C. C. Frederick, S. Y. Howell, Benjamin H. Grove, William C. Phelps, Bernhard Cohen, W. Scott Renner, John H. Pryor, Allen Jones, John H. Grant, T. H. McKee and A. T. Lytle.

On the arrival of the cortege at Forest Lawn, the Reverend Mr. Richards read the committal service, and so the earthly remains of Ernest Wende were delivered into the keeping of the Great Hereafter. Take him all in all we shall not look upon his like again.

## Figures and Tables

**Figure f1:**